# Characterisation of *Schizosaccharomyces pombe α*-actinin

**DOI:** 10.7717/peerj.1858

**Published:** 2016-03-28

**Authors:** Barbara Addario, Linda Sandblad, Karina Persson, Lars Backman

**Affiliations:** 1 Cell Biology Laboratory, School of Biochemistry and Cell Biology, BioScience Institute, University College Cork, Cork, Ireland; 2 Department of Molecular Biology, UmeåUniversity, Umeå, Sweden; 3 Department of Chemistry, Biological Chemistry, Umeå, Sweden

**Keywords:** Spectrin repeat, Actin-binding protein, α-actinin, *Schizosaccharomyces pombe*

## Abstract

The actin cytoskeleton plays a fundamental role in eukaryotic cells. Its reorganization is regulated by a plethora of actin-modulating proteins, such as a-actinin. In higher organisms, *α*-actinin is characterized by the presence of three distinct structural domains: an N-terminal actin-binding domain and a C-terminal region with EF-hand motif separated by a central rod domain with four spectrin repeats. Sequence analysis has revealed that the central rod domain of *α*-actinin from the fission yeast *Schizosaccharomyces pombe* consists of only two spectrin repeats. To obtain a firmer understanding of the structure and function of this unconventional *α*-actinin, we have cloned and characterized each structural domain. Our results show that this a-actinin isoform is capable of forming dimers and that the rod domain is required for this. However, its actin-binding and cross-linking activity appears less efficient compared to conventional *α*-actinins. The solved crystal structure of the actin-binding domain indicates that the closed state is stabilised by hydrogen bonds and a salt bridge not present in other *α*-actinins, which may reduce the affinity for actin.

## Introduction

In eukaryotes, the actin cytoskeleton plays important roles in most, if not all, cellular events, from motility to cell division. Its reorganization and dynamics are regulated by a variety of actin-modulating proteins, such as *α*-actinin, filamin, profilin and many more (Dos Remedios et al., 2003; McGough, 1998; Winder & Ayscough, 2005).

*α*-actinin, a ubiquitous actin cross-linking and bundling protein, belongs to the spectrin superfamily (Broderick & Winder, 2005; Djinovic-Carugo et al., 1999). This group of proteins is characterized by a highly conserved N-terminal actin-binding domain, a central rod domain consisting of spectrin-like repeats and a calmodulin-like domain at the C-terminal (Blanchard, Ohanian & Critchley, 1989; Otey & Carpen, 2004; Sjöblom, Salmazo & Djinovic-Carugo, 2008). As *α*-actinin forms antiparallel homodimers, it can cross-link actin filaments, thereby contributing to the three-dimensional organization of the actin cytoskeleton (Otto, 1994). Evolutionary studies of *α*-actinin have shown that the rod domain, essential for homodimer formation, is less well conserved compared to the other domains. In *Schizosaccharomyces pombe* (fission yeast) and all other fungi, the rod domain is predicted to contain only two, and not the usual four, spectrin-like repeats (Virel & Backman, 2004).

In vertebrates, except for birds there are four genes coding for four distinct *α*-actinins; two that that are calcium sensitive, present in non-muscle cells (*α*-actinin1 and *α*-actinin4) and two muscle isoforms that are calcium insensitive (*α*-actinin2 and *α*-actinin3) (Blanchard, Ohanian & Critchley, 1989; Burridge & Feramisco, 1981; Noegel, Witke & Schleicher, 1987). Skeletal muscle *α*-actinin isoforms are localized to the Z-disk (Luther, 1991), whereas non-muscle isoforms are localized to stress fibers, focal adhesion, neuronal synapsis and other structures (Lazarides & Burridge, 1975; Otey & Carpen, 2004; Walikonis et al., 2001). Fluorescence microscopy has localized *α*-actinin also to the cleavage furrows of chicken embryos (Fujiwara, Porter & Pollard, 1978) and sea urchin eggs (Mabuchi et al., 1985) suggesting a probable role of *α*-actinin in cytokinesis.

The fission yeast provides a simple eukaryotic model system to study the function of the actin cytoskeleton in cellular morphogenesis and cytokinesis (Chang & Nurse, 1996; Gould & Simanis, 1997; Naqvi et al., 1999). *S. pombe* is a unicellular archiascomycete, a subgroup of ascomycota, which shares many features with cells of more complex eukaryotes. The *S. pombe* genome was published in 2002 by the Wellcome Trust Sanger Institute (Wood et al., 2002). Comparison of gene sequences and phylogenetic analyses have suggested that the fission yeast diverged from budding yeast around 330–420 million years ago, and from metazoa and plants around 1,144 and 1,600 million years ago, respectively (Heckman et al., 2001; Sipiczki, 2000). Many of the *S. pombe* proteins have turned out to be more akin to their mammalian orthologs than to their *Saccharomyces cerevisiae* (budding yeast) counterparts, probably reflecting a more rapid evolution within fungal lineage than in metazoa (Doolittle et al., 1996; Sipiczki, 1995). In contrast to *S. pombe*, the genome of *S. cerevisiae* does not contain a gene coding for *α*-actinin (Virel & Backman, 2004).

In *S. pombe α*-actinin appears to be expressed only during cell division and then localizes mainly to the contractile ring in an actin-dependent manner (Coffman et al., 2009; Wu, Bahler & Pringle, 2001). Deletion of the gene (*ain1*) coding for *α*-actinin, does not interfere with normal growth of *S. pombe*. Under stress conditions deletion of *ain1* causes cytokinetic defects but still allow growth (Laporte et al., 2012; Wu, Bahler & Pringle, 2001). Based on further deletion experiments, it has been suggested that *α*-actinin and fimbrin have overlapping functions in cytokinesis (Wu, Bahler & Pringle, 2001). It appears that deletion of *ain1* does not affect any other cellular process.

It is general believed that a major function of *α*-actinin is to cross-link actin filaments into bundles or networks. However, it has been suggested that in contrast to other *α*-actinins, the paralogue of *S. pombe* binds actin weaker and is a less efficient cross-linker (Li, 2014). To better understand the role of *α*-actinin in cytoskeletal organization we have cloned, expressed and characterized the structural domains of this unconventional *α*-actinin.

## Materials and Methods

### Cloning, expression and purification

Genomic DNA coding for *S. pombe α*-actinin was obtained from a cosmid library (Wellcome Trust Sanger Institute, Hinxton, UK) by PCR using primers 5′-ATGCAGG CAAATCAATGGCAAA-GCG and 5′-TTAAACTATTTCTTTGTCTTCGGCCAG and inserted into the TA-cloning vector, pTZ57R/T (Fermentas, Leon-Rot, Germany). The gene contained two introns, 51 and 140 nucleotides long, respectively. These introns were removed by deletion mutation using QuikChange Site-Directed Mutagenesis kit (Stratagene). For this purpose, two primer pairs were designed (Intron I: 5′-AAATGG TTCAACACAAAACTTTCATCGAGAGACT and 5′-GTGTTGAACCATTTTGTAAATG TTCTATT; Intron II: 5′-GGTCCTGCTGATATTGTG-GATGGGAACCTGA and 5′-CCC ATCCACAATATCAGCAGGACCAATGTTGGTC). In both case, the reaction mixtures were prepared essentially according to the manufacturer’s instructions.

To delete the 51-bp intron, an initial 30 s denaturation step at 95°C was followed by 18 cycles of 30 s at 95°C, 60 s at 50°C and 7 min at 68°C. The cycling parameters to delete the other intron were the same except that denaturation was followed by 60 s at 54°C and that 20 cycles were run. To digest parental methylated DNA DpnI was added directly to the reaction mixture and incubated for 2 h at 37°C. To improve transformation efficiency, amplified DNA was ligated before heat-shock transformation.

This plasmid with the intron-free insert was then used as template together with primers 5′-TTTGGATCCATGCAGGCAAATCAATGGCAAAGCG and 5′-TTTCTCGAGTTAAACT ATTTCTTTGTCTTCGGCC in another PCR amplification. The amplified PCR product was digested by BamHI and XhoI (underlined) and ligated into pET-TEV (a modified pET-19b vector) containing an N-terminal 10xHis-tag and a TEV protease cleavage site.

Upon sequencing (carried out by Eurofins MWG GmbH, Ebersberg, Germany), it turned out that the intron-free *S. pombe α*-actinin gene contained seven point mutations and one deletion compared to the reference sequence (NM_001019718). The cloned gene was corrected by site-directed mutagenesis, using the following primer pairs: Insertion and mutation I: Forward 5′-GGTTCAACACAAAACTTTCATCGAGAGACTTACCATCTGTGT TTGACTTGAGAAAGG and Reverse 5′-CCTTTCTCAAGTCAAACACAGATGGTAAGTCT CTCGATGAAAGTTTTGTGTTGAACC; mutation II: Forward 5′-GCTTTAGAATATATAAA AAGCAAAGGAATGCCGTTGACCAACATTGG and Reverse 5′-CCAATGTTGGTCAA CGGCATTCCTTTGCTTTTTATATATTCTAAAGC; mutation III: Forward 5′-GATTTT ACTCGGAGTTGGACAAACGGCTTG and Reverse 5′-CAAGCCGTTTGTCCAACTCCG AGTAAAATC; mutation IV: Forward 5′-CCAATTTACAGGGTGATTGGCGTGACCAA CTCGACC and Reverse 5′-GGTCGAGTTGGTCACGCCAATCACCCTGTAAATTGG; mutation V: Forward 5′-CCAATATCATTGCCAATAAGATCAAGTACCTTGAGAATG and Reverse 5′-CATTCTCAAGGTACTTGATCTTATTGGCAATGATATTGG; mutation VI: Forward 5′-GGTTCGTCCTAATATAGTAAAGTTTTTAGAATGCAACATGAAC and Rev 5′-GTTCATGTTGCATTCTAAAAACTTTACTATATTAGGACGAACC. To improve the amplification DMSO was added to the sample reaction and after the last PCR cycle an extra step at 72°C for 10 min was included to ensure that any remaining single-stranded DNA was fully extended. GeneScript (USA) corrected the final mutation as well as controlled the fidelity of the final clone (pTEV-SP).

The database SUPERFAMILY was used to determine breakpoints for the structural domains of *S. pombe α*-actinin (Wilson et al., 2009). Gene fragments coding for the structural domains were excised by PCR using the intron- and mutation-free clone as template. The actin-binding domain (ABD), spanning nucleotides 1–702 (residues 1–234) was amplified using primers 5′-TTTGGATCCATGCAGGCAAATCAATGGCAAAGCG and 5′-TTTCTCGAGTTATTTATCCAAGGTAGAAAACGC, the rod domain containing two putative spectrin repeats (ROD), spanning nucleotides 682–1449 (residues 228–483) was amplified using primers 5′-TTTGGATCCGCGTTCTACCTTGGAT-AAAGTGG and 5′-TTTCTCGAGTTATTTGGAGAGTGTTCTCTTTTC, the calcium-binding domain (EF), spanning nucleotides 1429–1863 (residues 477–621) was amplified using primers 5′-TTTGGATCCGAAAAGAGAACACTCTCCAAAC and 5′-TTTCTCGAGTTAAACTATTT CTTTGTCTTCGGCC. The ABD-ROD (nucleotides 1–1449, residues 1–483) and ROD-EF (nucleotides 682–1863, residues 228–621) were amplified using the proper primers. The BamHI and XhoI restriction sites (underlined) were used to ligate amplified PCR products into pET-TEV, giving rise to plasmids pTEV-SP-ABD, pTEV-SP-ROD, pTEV-SP-ABD-ROD, pTEV-SP-ROD-EF, pTEV-SP-ROD-EF and pTEV-SP-EF. The gene fragment coding for the calcium-binding domain was also inserted into the plasmid pGEX-6-P and subsequently a 6xHis-tag was added to the C-terminal to improve purification, giving the plasmid pGST-TEV-SP-EF-His.

**Figure 1:**
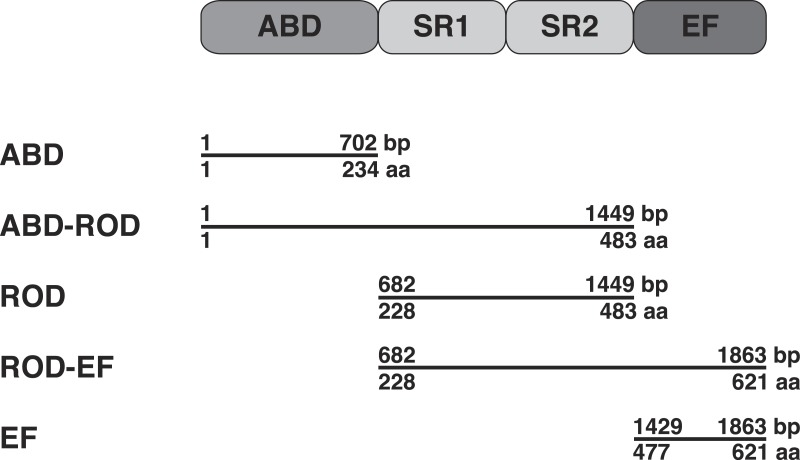
Domain organization of *S. pombe α*-actinin and expressed peptides. The full-length protein contains a N-terminal actin-binding domain (ABD), a central rod (ROD) domain composed of two spectrin repeats (SR1 and SR2) and a C-terminal calcium-binding domain (EF) (bp, base pair; aa, amino acid residues).

The correctness of inserted gene fragments was verified by sequencing (Eurofins MWG GmbH, Ebersberg, Germany). Figure 1 shows the domain organization of *S. pombe α*-actinin.

*E. coli* BL21(DE3) cells were transformed (by heat-shock) with the purified plasmids containing the different constructs. The transformed cells were cultured at 37°C in Luria-Betani medium containing 100 µM carbenicillin until an optical density of 0.6–0.8 at 600 nm was reached. Protein expression was induced by addition of isopropyl thio-*β*-D- galactoside to a final concentration of 0.5 mM and cells were grown overnight at 23°C. Cells were harvested by centrifugation (29,000× g for 15 min), resuspended in 25 mM sodium phosphate buffer, pH 7.6, 150 mM NaCl, 10 mM imidazole, 1% Triton X-100 and then lysed by sonication. Cell debris was removed by centrifugation at 37,000× g for 20 min and the clarified supernatant loaded onto a HiTrap^TM^ Chelating HP column (GE Healthcare Bioscience AB, Lund, Sweden) charged with nickel. Unbound proteins were eluted with 25 mM sodium phosphate buffer, pH 7.6, 150 mM NaCl, 10 mM imidazole. Bound proteins were eluted with an imidazole gradient ranging from 10 to 510 mM imidazole in the same buffer. Before addition of Tobacco Etch Virus (TEV) protease (kindly provided by Dr. David S. Waugh), imidazole was removed by gel filtration on HiPrep desalting columns (GE Healthcare Bioscience AB, Lunds, Sweden). The released 10xHis-tag and the 6xHis-tagged TEV protease were removed by affinity chromatography as before. When necessary, remaining impurities were removed by ion exchange chromatography on a HiTrap Q column (GE Healthcare, Sweden). The target protein was eluted using a salt gradient (1 M NaCl) in 50 mM Tris–HCl, pH 7.6. Finally, purified polypeptides were transferred into either 25 mM sodium phosphate buffer, pH 7.6, 150 mM NaCl or, to improve solubility, 50 mM Tris, pH 7.6, 200 mM KCl by gel filtration on HiPrep desalting columns (GE Healthcare Bioscience AB, Sweden).

The clarified lysate of the EF polypeptide was loaded on a Glutathione-Sepharose column (GE Healthcare Bioscience AB, Uppsala, Sweden). After unbound proteins had been eluted bound proteins were eluted with 10 mM glutathione in 25 mM sodium phosphate buffer, pH 7.6, 150 mM NaCl. The GST-tag was liberated by overnight incubation in the presence of TEV protease. The GST-tagged moiety was removed by a second passage over the Glutathione-Sepharose. To remove remaining impurities, the GST-free EF polypeptide was purified using affinity chromatography on a HiTrap™ Chelating HP column as described above. The EF polypeptide was transferred into 50 mM Tris, pH 7.6, 200 mM KCl by gel filtration on a Hiprep 26/10 desalting column (GE Healthcare Bioscience AB, Uppsala, Sweden).

**Figure 2:**
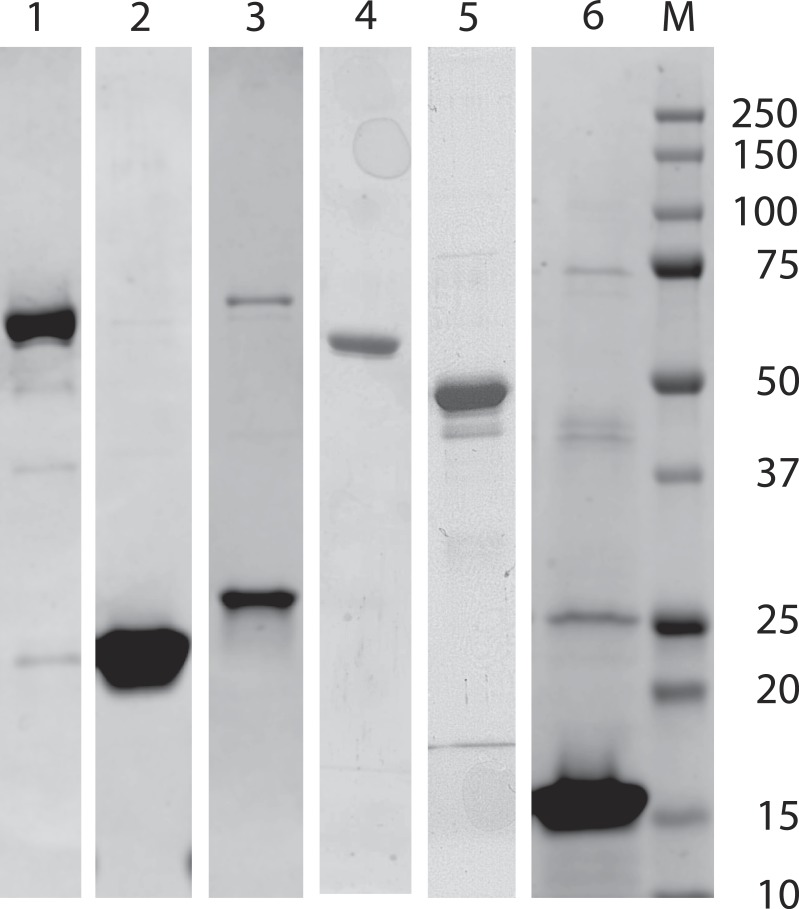
SDS-PAGE analysis of expressed and isolated recombinant polypeptides. Lane 1: full-length *S. pombe α*-actinin; lane 2: ABD; lane 3: ROD; lane 4: ABD-ROD; lane 5: ROD-EF; lane 6: EF-his; lane M: molecular weight markers: 250, 150, 100, 75, 50, 37, 25, 20, 15 and 10 kDa.

Protein concentration was determined from the absorbance at 280 nm using the molar absorptivity, as calculated from the amino acid sequence (using ProtParam at the ExPASy proteomics server). The purity of the expressed polypeptides was routinely determined under denaturating conditions by SDS-polyacrylamide gel electrophoresis (Laemmli, 1970) (Fig. 2).

### Gel filtration

Gel filtration in 25 mM sodium phosphate buffer, pH 7.6, 150 mM NaCl, on a Sephacryl S-400HR column (0.66 × 37 cm, GE Healthcare Bioscience AB, Uppsala, Sweden) and in 50 mM Tris–HCl buffer, pH 7.6, 200 mM KCl, on a Superdex^TM^ 200 10/300GL column (GE Healthcare Bioscience AB, Uppsala, Sweden) were used to determine the native molecular size of purified polypeptides. Thyroglobulin (669 kDa, Stokes radius: 8.58 nm), ferritin (440 kDa, Stokes radius: 6.10 nm), aldolase (158 kDa, Stokes radius: 4.81 nm), *γ*-globulin (158 kDa, Stokes radius: 5.22 nm), bovine serum albumin (67 kDa, Stokes radius: 3.55 nm), ovalbumin (44 kDa, Stokes radius: 2.80 nm), chymotrypsinogen (25 kDa, Stokes radius: 2.09 nm) and myoglobulin (17 kDa, Stokes radius: 1.90 nm) were used as molecular size references. It should be noted that bovine serum albumin forms dimers and tetramers that give rise to noticeable peaks in the elution profile that can be used as references.

### Actin co-sedimentation assay

Binding to actin was determined by a co-sedimentation assay using human platelet non-muscle actin (Cytoskeleton, Denver, CO, USA). Actin dissolved in 5 mM Tris–HCl, pH 8.0, 0.2 mM CaCl_2_ and 0.2 mM ATP was polymerised by addition of KCl and MgCl_2_ to final concentrations of 50 mM and 2 mM, respectively. After 1 h at room temperature, the polymerised actin was mixed with varying concentrations of either full-length *α*-actinin or ABD. After incubation for 30 min at room temperature the reaction mixture was centrifuged at 13,000 rpm (16,000× g) for 15 min (bundling assay) or at 90,000 rpm (350,000× g) for 60 min (binding assay). Supernatants and pellets were separated and analysed by SDS-PAGE.

### Negative staining electron microscopy

Samples for electron microscopy were prepared by mixing polymerised human platelet non-muscle actin (5 µM) with varying concentrations of full-length *α*-actinin and incubated for at least 30 min at room temperature. For negative staining copper grids coated with formvar and carbon, were prepared with Leica EM ACE200 carbon coating system. The grids were glow-discharged with Pelco easiGlow system, (Ted Pella, Inc.) prior to adsorption of 3.5 µl of assembled filaments for 2 min, washed two times in H_2_O and immediately negatively stained in 50 µl of 1.5% uranyl acetate solution for 30 s. Negative stained samples were examined with a JEOL 1230 transmission electron microscope operating at 80 kV at 5,000×, 10,000× and 80,000× magnifications. Micrographs were recorded with Gatan 830 SC200 Orius charge-coupled device camera, with 2 k × 2 k pixels, using the Digital Micrograph software.

### Circular dichroism (CD) spectroscopy

The folding of expressed polypeptides in 25 mM sodium phosphate buffer, pH 7.6, 150 mM NaCl, was analysed by CD spectroscopy using a Jasco J-810 spectrometer. Spectra between 200 and 260 nm were collected at 2°C using 0.025 nm step-size and a scan speed of 20 nm per min. Mean residue molar ellipticity was calculated from three accumulated spectra.

### Crystallisation

Initial crystallization trials of ABD were performed by the sitting-drop vapour-diffusion method in a 96-well MRC-crystallization plate (Molecular Dimensions). Droplets of 0.5 µL protein solution (23 mg ml^−1^) were mixed with an equal volume of reservoir solution using screens from Hampton Research (Crystal Screen HT) and Molecular Dimensions (PACT I + II). Crystals were obtained from similar crystallization solutions in the two screens, #G3 in Crystal Screen HT and #B12 in the PACT I + II screen. Optimized crystals were obtained by mixing the protein with 0.1 M MES pH 6.5, 5 mM ZnCl_2_ and 18% PEG 6000 in equal volumes and incubated on ice for 30 min before the solution was spun. Drops (4 µl) were allowed to equilibrate against the well solution as hanging drops. Crystals grew within a few days and contained one molecule in the asymmetric unit (*V*_*M*_ = 2.31Å^3^Da^−1^) in space group P2_1_2_1_2_1_ with unit cell dimensions *a* = 33.54 Å *b* = 79.47 Å and *c* = 91.93 Å.

### Data collection and structure determination

Crystals were flash-cooled in liquid nitrogen after a 20 s soak in the crystallization solution supplemented with 15% glycerol. X-ray diffraction data to 1.46 Å resolution were collected at beamline ID29 at the European Synchrotron Radiation Facility, ESRF, in Grenoble, France using a Pilatus 6M detector (Dectris). Data were processed with XDS (Kabsch, 2010) and scaled with AIMLESS (Evans & Murshudov, 2013) from the CCP4 program suite. The structure of ABD was solved with molecular replacement using PHASER (McCoy et al., 2007) with the human *α*-actinin ABD as search model (PDB code 2EYI). The model was manually rebuilt and refined using iterative cycles of Coot (Emsley et al., 2010) and PHENIX (Adams et al., 2010; Adams et al., 2002). In the last rounds of refinement translational-libration-screw (Painter & Merritt, 2006; Winn, Isupov & Murshudov, 2001) refinement was used, treating each domain as an individual TLS group. Hydrogen atoms were included and refined in the final model. The quality of the model was analysed with MolProbity in PHENIX (Chen et al., 2010) Statistics for data collection, processing and refinement are summarized in Table 1. Figures were drawn with CCP4MG (McNicholas et al., 2011) and Chimera (Pettersen et al., 2004). The X-ray coordinates and structure factors have been deposited in the Protein Data Bank under pdb ID: 5BVR.

**Table 1:** Processing and refinement statistics. Values in parentheses indicate statistics for the highest resolution shell.

**Data collection**	
Space group	*P*2_1_2_1_2_1_
Cell dimensions a, b, c (Å)	33.54 79.47 91.93
Wavelength (Å)	0.972
Resolution (Å)	45.97–1.46
Highest resolution shell (Å)	1.54–1.46
*R*_merge_^a^ (%)	6.3 (81.2)
*R*_pim_^b^ (%)	2.8 (34.3)
Total number of observations	309519 (44725)
Unique reflections	43523 (6161)
*I*∕*σ* (I)	14.3 (2.1)
Completeness (%)	99.6 (98.5)
*CC*_1∕2_^c^	0.999 (0.797)
Multiplicity	7.1 (7.3)
Wilson B-factor (Å^2^)	15.4
**Refinement**	
Resolution (Å)	45.97–1.46 (1.51–1.46)
Rwork^d^ (%)	15.79 (26.11)
Rfree^d^ (%)	18.66 (28.41)
RMSD from ideal	
Bond lengths (Å)	0.009
Bond angles (°)	1.15
Average B-factor (Å^2^)	26.7
Protein	25.1
Zinc ions	21.7
Water	37.5
Ramachandran plot (%)	
Favoured, allowed, outlier	97.0/ 3.0/ 0

**Notes.**

a *R*_merge_ = Σ_*hkl*_Σ_*i*_|*I*_*i*_(*hkl*) − 〈*I*(*hkl*)〉|∕Σ_*hkl*_Σ_*i*_*I*_*i*_(*hkl*), where *I*_*i*_(*hkl*) is the intensity of the *i*th observation of reflection *hkl* and 〈*I*(*hkl*)〉 is the average over of all observations of reflection *hkl*.

b *R*_pim_ = precision-indicating merging *R* factor (Weiss, 2001).

c *CC*_1∕2_ is the correlation between intensities from random half data sets (Karplus & Diederichs, 2012).

d *R*_work_ = Σ||*F*_obs_| − |*F*_calc_||∕Σ|*F*_obs_|, where *F*_obs_ and *F*_calc_ are the observed and calculated structure factor amplitudes, respectively. *R*_free_ is equivalent to *R*_work_ but is calculated using a 5% randomly selected set of reflections which is excluded from refinement.

## Results and Discussion

### Cloning, expression and purification

We have cloned full-length *α*-actinin from a cosmid library of *S. pombe*. For this purpose, PCR was used to amplify the *α*-actinin gene from the library. The PCR product was then subcloned into a TA-vector that facilitates ligation of DNA fragments produced by TAQ polymerase. In a second PCR, the TA-vector with insert was used as template for amplifying the *α*-actinin gene with suitable restriction sites for cloning into the expression vector, pET-TEV.

The isolated *α*-actinin gene contained two introns, 51 and 140 nucleotides long, respectively. These introns were removed by deletion mutations, resulting in an intron-free plasmid, pTEV-SP. However, it turned out that the sequence contained seven mutations (all causing an amino acid residue change) and one deletion (creating a premature stop codon) compared to the reference sequence (NM_001019718). Therefore we used several consecutive rounds of site-specific mutagenesis to correct these mutations.

Upon expression and purification of the *α*-actinin it was apparent that the solubility of the full-length protein was low; with time it precipitated when the imidazole used to elute it from the affinity column was removed. We decided therefore to express each domain separately as well as in pairs instead. All constructs expressed reasonable levels of protein, although the solubility differed. All but ABD and EF precipitated with time when stored in sodium chloride containing buffers. By changing to potassium chloride the solubility increased significantly but still precipitation occurred with time, particular at lower temperatures.

All expressed polypeptides could be easily purified by affinity and (when needed) ion exchange chromatography to high purity (Fig. 2). The sizes of these polypeptides as estimated from Coomassie blue-stained SDS-polyacrylamide gels were in good agreement with calculated molecular weights (Table 2).

**Table 2:** Properties of expressed peptides.

Domain	Size			[Θ_222_]_MRW_ degrees cm^2^ dmol^−1^ residue^−1^	*α*-helix^c^ %	*α*-helix^d^ %	Dimer formation
	Residues^a^	Calculated (kDa)	SDS-PAGE (kDa)^b^				
ABD	236	27.4	26	15,053	46.3	47.5	no
ROD	258	30.4	30	18,060	54.0	81.0	yes
EF	147	16.9	17	12,919	40.8	61.2	no
EF-his^e^	156	18.0	17	nd	nd	nd	no
ABD-ROD	485	56.7	57	20,807	61.0	66.6	yes
ROD-EF	396	46.3	47	24,351	70.1	78.0	yes

**Notes.**

a Includes two N-terminal residues from the cloning vector, except for EF-his that includes 3 N-terminal residues and 6 histidines at the C-terminal.

b Estimated from Coomassie blue-stained polyacrylamide gels.

c The *α*-helical content estimated as: (−[Θ_222_]_MRW_ + 3,000)∕39,000 (Morrow et al., 2000).

d MLRC (Guermeur et al., 1999) at Pôle Bioinformatique Lyonnaise was used to predict the secondary structure.

e EF-his was obtained from plasmid pGST-TEV-SP-EF.

nd Not determined

However, shortly after imidazole was removed from the ABD-ROD two bands around 28 kDa were seen on stained gels. Upon storage, these bands grow in intensity at the same time as the initial band at 57 kDa diminished. To our surprise, LC-MS/MS analysis indicated that both bands around 28 kDa comprised the N-terminal part of ABD-ROD, with a sequence coverage up to residue 240 in both cases. When loading less protein on the gel, a band around 55 kDa was clearly seen. Therefore it is likely that continuous degradation of the 57 and 55 kDa bands gave rise to the doublet around 28 kDa (not shown). It is tempting to assume that the cleavage site is somewhere in the neck region that connects the actin-binding domain with the rod domain. In vertebrate *α*-actinins, this region is known to be susceptible to proteolytic cleavage (Sjöblom, Salmazo & Djinovic-Carugo, 2008).

**Figure 3:**
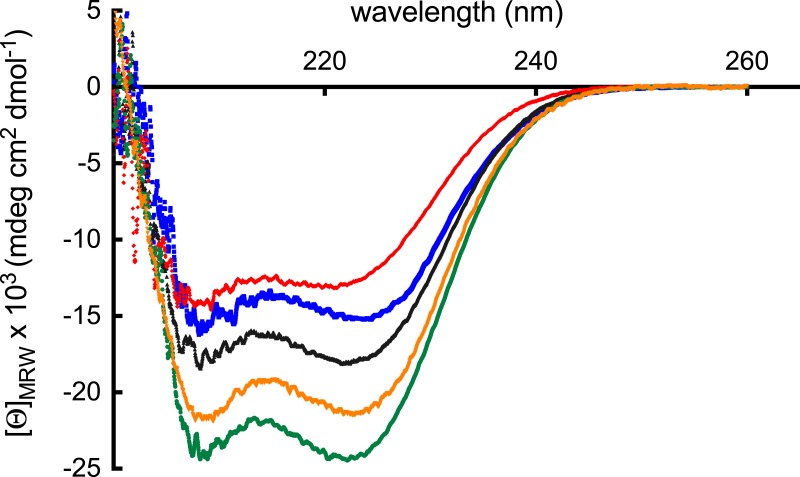
Far-UV CD spectra. The far-UV spectra of 7.7 µM ABD (blue), 11.3 µM ROD (black), 21.6 mM EF (red), 2.2 µM ABD-ROD (gold) and 7.7 µM ROD-EF (green) of *S. pombe α*-actinin in 25 mM sodium phosphate, pH 7.6, 150 mM NaCl. The mean residue molar ellipticity was determined from 3 accumulated scans between 200 and 260 nm, at 2 °C.

CD measurements in the far UV region showed that all five polypeptides displayed negative peaks at 208 and 222 nm, respectively (Fig. 3). Since this is a typical characteristic of proteins rich in *α*-helices, the results indicated that all polypeptides were folded.

### Dimer formation

**Figure 4:**
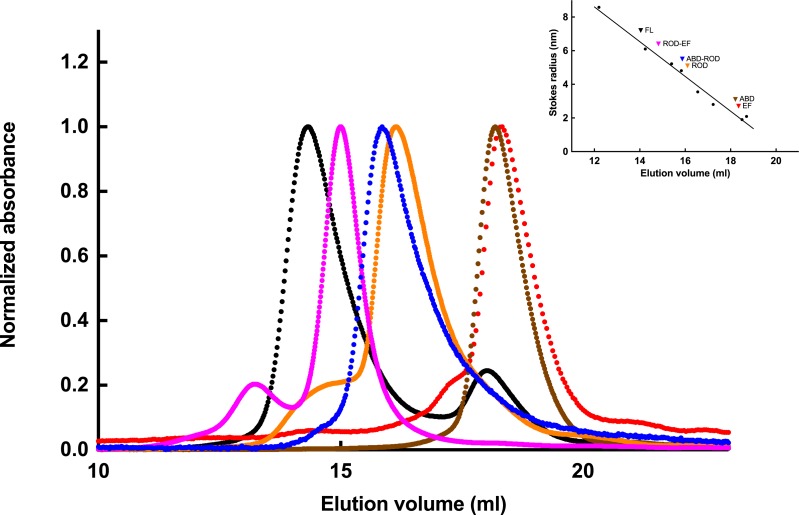
Gel filtration of expressed *S. pombe* peptides. The apparent molecular size of ABD (brown), ABD-ROD (blue), ROD (gold), ROD-EF (magenta) and EF (red) as well as of full-length *α*-actinin (black) were determinate by gel filtration on a Superdex™ 200 10/300GL, equilibrated with 50 mM Tris–HCl, pH 7.6, 200 mM KCl. Elution patterns were determined by absorbance at 280 nm. Inset. Stokes radii were determined using thyroglobulin (669 kDa, 8.58 nm), ferritin (440 kDa, 6.10 nm), aldolase (158 kDa, 4.81 nm), *γ*-globulin (158 kDa, 5.22 nm), bovine serum albumin (67kDa, 3.55 nm), ovalbumin (44 kDa, 2.80 nm), chymotrypsinogen (25 kDa, 2.09 nm) and myoglobulin (17 kDa, 1.90 nm) as molecular size references.

The ability of each expressed polypeptide to form dimers was investigated by gel filtration. Figure 4 shows the elution profiles of the polypeptides. When these profiles were compared to a set of reference proteins, it was obvious that only ABD and EF eluted as expected from the calculated molecular weight (27.4 and 18.0 kDa, respectively).

Since full-length *α*-actinin as well as any polypeptide containing the rod domain will have an elongated shape, it was expected that this would give rise to anomalous elution profiles. The full-length *α*-actinin eluted earlier than a much larger protein, ferritin (440 kDa). The determined hydrodynamic (Stokes) radius was 6.3 nm, which compared favourably with the radius of *Entamoeba histolytica α*-actinin2 (6.1 nm) that is of similar size and also has a rod domain containing two spectrin repeats (Addario & Backman, 2010).

The ROD-EF (46.3 kDa) eluted close to a nearly ten times larger protein, ferritin (440 kDa). The determined Stokes radius of ROD-EF was 5.7 nm, which compared favourably with the radius of a similar polypeptide of *E. histolytica α*-actinin2 (5.7 nm) (Addario & Backman, 2010). However, the determined Stokes radii of *S. pombe* ROD (4.4 nm) and ABD-ROD (4.6 nm) were smaller than those of the corresponding and similar sized *Entamoeba* polypeptides (5.2 and 5.4 nm, respectively) but still larger than the nominal sizes.

Since polypeptides including the rod domain, behaved as larger molecules than expected, even for an elongated molecule, it is apparent that this structural domain is essential for dimer formation (or oligomers) also in this *α*-actinin, similar to all other isoforms studied (Blanchard, Ohanian & Critchley, 1989; Kahana & Gratzer, 1991; Ylänne et al., 2001).

### Actin binding

In order to cross-link or bundle actin filaments, two discrete actin-binding sites must be available in the cross-linking protein. Alternatively, a dimer (or higher oligomer) with a single site in each monomer would also suffice. Since monomeric *α*-actinin contains a single actin-binding domain, it would be necessary for *α*-actinin to form dimers for cross-linking activity. Therefore it was assumed that only ABD and ABD-ROD would bind actin filaments and that only the ABD-ROD polypeptide would cross-link or bundle actin filaments in addition to the full-length *α*-actinin.

**Figure 5:**
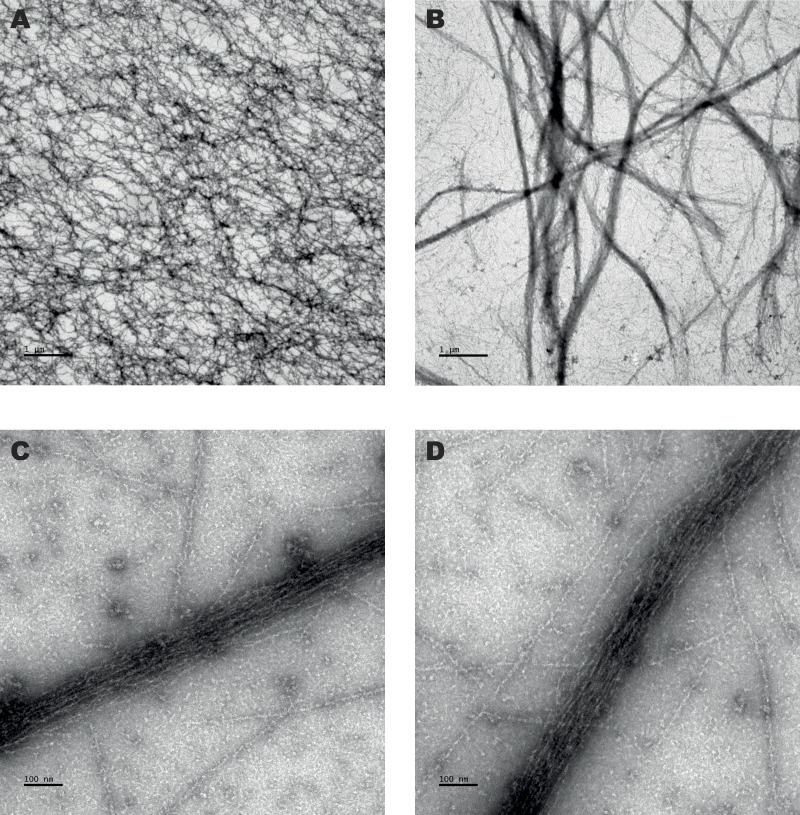
Negative staining electron microscopy. 5 µM actin was incubated alone (A) or with 2.4 µM (B) and 3.7 µM (C and D) *S. pombe* full length *α*-actinin before staining, as described in Material and Methods. Samples were adsorbed onto grids and negatively stained with 1.5% uranyl acetate. Scale bar: 1 µm (A and B) and 100 nm (C and D).

**Figure 6:**
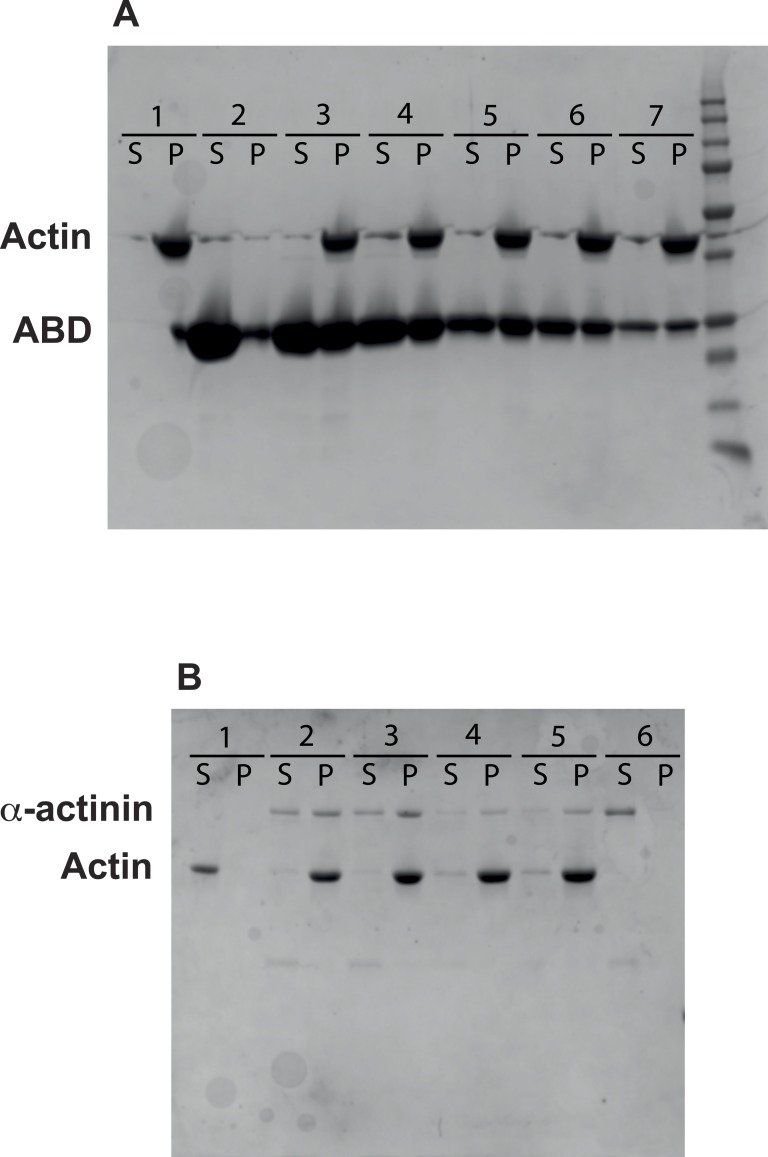
Actin binding and bundling. (A) Human platelet non-muscle actin was mixed with varying concentrations of ABD, incubated at room temperature for 60 min and centrifuged for 60 min@ 90,000 rpm. Supernatants and pelleted proteins were analysed by SDS-PAGE. Lane 1: 12 µM actin; lane 2: 251 µM ABD; lane 3: 12 µM actin and 251 µM ABD; lane 4: 12 µM actin and 126 µM ABD; lane 5: 12 µM actin and 63 µM ABD; lane 6: 12 µM actin and 31 µM ABD; lane 7: 12 µM actin and 16 µM ABD. (B) Actin was mixed with full length *α*-actinin, incubated as before and centrifuged for 11 min@ 13,000 rpm. Supernatants and pelleted proteins were analysed by SDS-PAGE. Lane 1: 5 µM actin; lanes 2 and 3: 5 µM actin and 3.7 µM *α*-actinin; lanes 4 and 5: 5 µM actin and 1.2 µM *α*-actinin; lane 6: 3.7 µM *α*-actinin.

As can be seen in Fig. 5, electron microscopy showed bundle-formation in the presence of full-length *α*-actinin. To verify that full-length *α*-actinin cross-links or bundles actin filaments we used an actin sedimentation assay (Addario & Backman, 2010). In this assay, a low-speed centrifugation should pellet the cross-linker if actin networks and/or bundles are formed after incubation of the cross-linker with actin filaments. Similarly, a high-speed centrifugation should pellet any actin-binding protein together with actin filaments. Therefore varying concentrations of full-length *α*-actinin or ABD was incubated with non-muscle actin filaments before centrifugation. As expected, in the presence of full-length *α*-actinin, actin filaments could be pelleted by a low-speed centrifugation (Fig. 6). Moreover, after a high-speed centrifugation, actin filaments together with ABD were detected in the pellet, indicating a direct binding. It is evident that *S. pombe α*-actinin dimerizes as well as cross-links or bundles actin filaments as all other studied *α*-actinins.

When non-muscle actin was exchanged for rabbit skeletal muscle actin in the assay, we could not detect any bundling or binding activity (not shown). Although we could observe some bundle formation by electron transmission microscopy even in this case, it was apparent that it was not as extensive as observed for non-muscle actin. This is in accordance with previous suggestion that *S. pombe α*-actinin is a less efficient cross-linker of muscle actin (Li, 2014).

**Figure 7:**
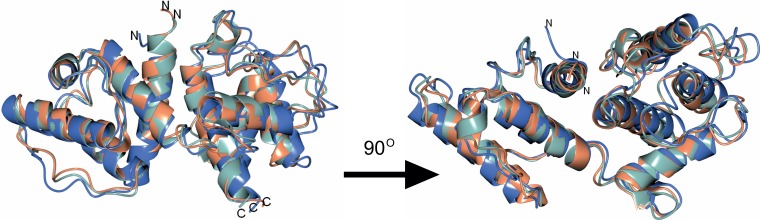
Structures of *S. pombeα*-actinin and human *α*-actinins 3 and 4. The structure of the actin-binding domain of *S. pombe* was superimposed with human *α*-actinin 3 (pdb ID: 1WKU) and 4 (pdb ID: 2R0O). *S. pombe α*-actinin, 1WKU and 2R0O are depicted in blue, see green and coral, respectively.

**Figure 8:**
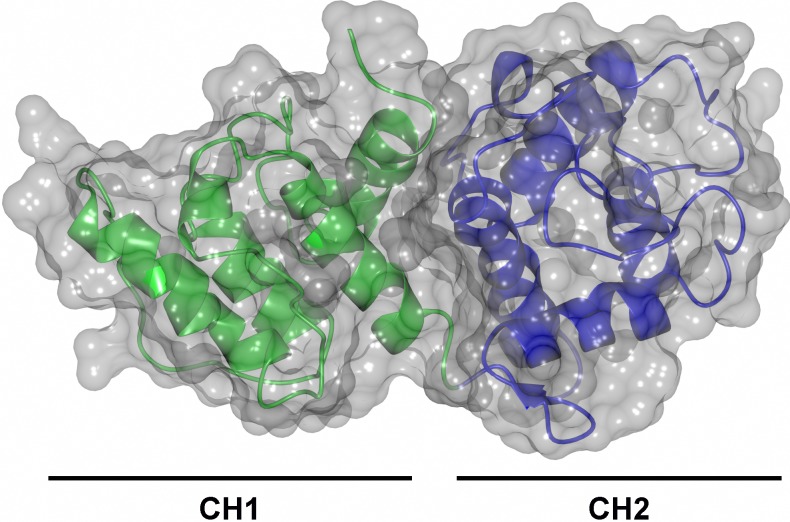
Schematic view of the *S. pombe α*-actinin actin-binding domain. The CH1 domain (residues 1–119) is depicted in green while the CH2 domain (residues 120–254) is depicted in blue. The shape of the protein is shown as a transparent surface.

### Structure of ABD

The sequence of *S. pombe* ABD is ca 50% identical to the actin-binding domain of human *α*-actinin and ca 40% to that of human plectin. However, when the Dali server (Holm & Rosenstrom, 2010) was interrogated several structures were returned with very high Z-scores and low root mean deviations (rmsd). For instance, the returned Z-score and rmsd of the calcium-insensitive human *α*-actinin3 (pdb ID: 1WKU) and the calcium-sensitive *α*-actinin4 (pdb ID: 2R0O) were 32.5 and 1.3 Å, and 31.5 and 1.4 Å, respectively. Indeed, the overall fold of *S. pombe α*-actinin ABD is very similar to other actin-binding domains. The major structural differences are located to loops connecting the helices (Fig. 7).

**Figure 9:**
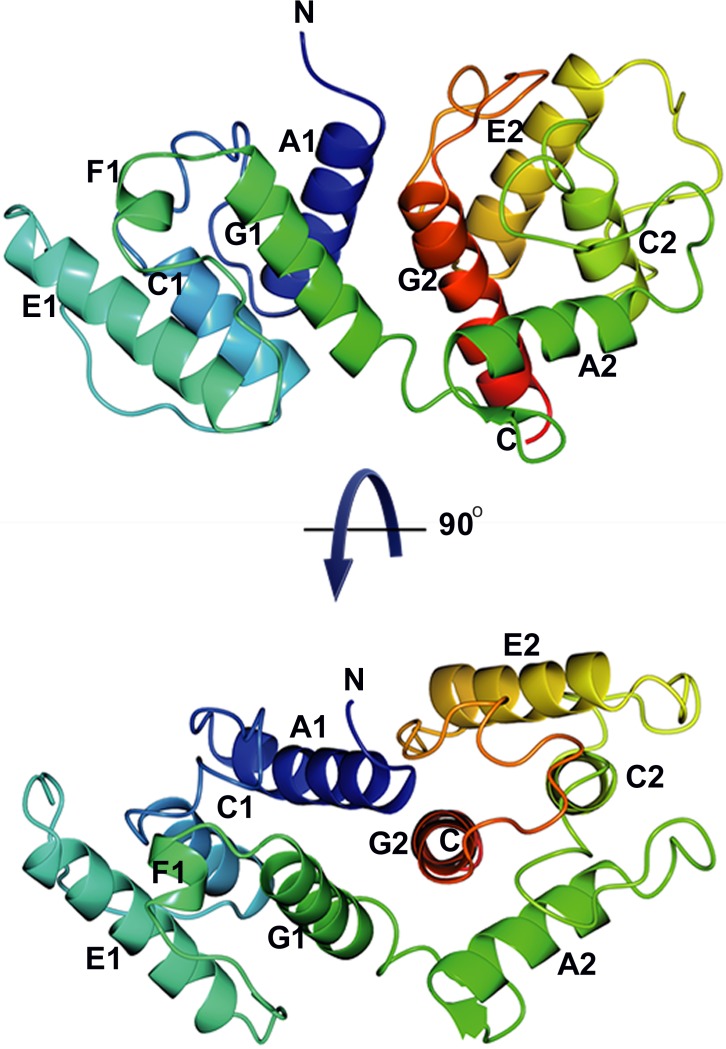
Overall structure of *α*-actinin ABD. The coloring blends through the model from blue (N-terminus) to red (C-terminus). The model is shown in two directions, rotated 90 degrees with respect to each other.

The structure of *S. pombe* ABD consists of two calponin homology domains with similar folds, CH1 (residues 6–119) and CH2 (residues 120–234) (Fig. 8). The domains share 17% sequence identity calculated on 99 structurally equivalent residues (rmsd = 2.0 Å) as calculated with Dali (Holm & Rosenstrom, 2010). Each domain consists of four long helices A1, C1, E1, G1 and A2, C2, E2 and G2 respectively (Fig. 9). The naming of the helices is based on the human *α*-actinin ABD (Borrego-Diaz et al., 2006). The main differences between CH1 and CH2 are located to the loops connecting the helices. For instance, in CH2 a short *β*-hairpin precedes helix A2 and in the segment connecting helices E1 and G1 in CH1 a small helix, F1, is located, a region that in the CH2 domain is mainly coiled.

**Figure 10:**
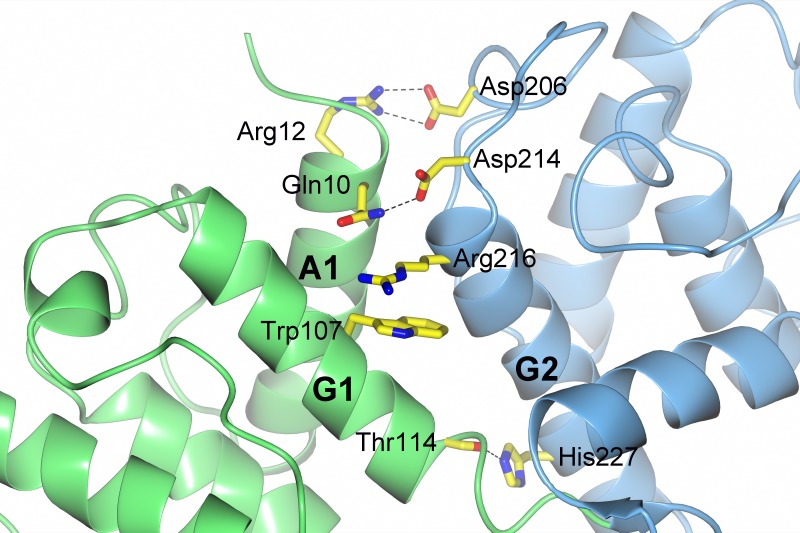
Interactions between the CH1 and CH2 domains. Hydrogen bonds (dashed lines) are formed between Arg12 and Asp206, Gln10 and Asp214, and between Thr114 and His227. Trp107 and Arg216 stack and form a *π*-cation interaction.

The structure was solved in the closed conformation where the interactions between the CH1 and CH2 domains are mainly caused by helices A1 and G1 (CH1 domain) and helix G2 and the coiled region between helices E2 and G2 (CH2 domain). The hydrogen bonding pattern between CH1 and CH2 is very much conserved when comparing *S. pombe* ABD with other ABDs. The similarities are for instance a hydrogen bond between Gln10 and Asp214 located on A1 and G2 respectively and between the carbonyl oxygen of Thr114 (G1) and the side chain of His227 (G2). Further, there is a *π*-cation interaction between two residues from G1 and G2. In the *S. pombe* protein this interaction occurs between the conserved Trp107 and Arg216. The position equivalent of Arg216 is occupied by a lysine in most other structures. As calculated by the CaPTURE server (Gallivan & Dougherty, 1999) this Trp-Arg interaction is listed as energetically significant (electrostatic energy −1.9 kcal/mol) whereas a Trp-Lys interaction is not (electrostatic energy >−1.0 kcal/mol). This implies a stronger interaction between the domains in the *S. pombe* protein. In addition, there is a salt bridge present between helix A1 in CH1 and the loop between E2 and G2 in CH2 in the *S. pombe* protein; a strong bidentate hydrogen bond interaction between Arg12 and Asp206 (hydrogen bond distances: 2.9 and 3.1 Å, respectively). This interaction has no equivalent interaction in the human ABD. However, in mouse plectin (pdb ID: 1SH5) a similar bidentate interaction is found between Arg11 and Asp214 (hydrogen bonding distance: 2.8 and 3.5 Å, respectively). Taken together, the strength of the *π*-cation bond between Trp107 and Arg216 and the interaction between Arg12 and Asp206 indicate strong interactions between CH1 and CH2 and may favor a closed conformation of the actin-binding domain (Fig. 10). An additional difference between the *S. pombe* ABD and the human ABDs is the interaction involving the coil between helix E2 and G2 and the coiled region between A2 and C2. In human ABD (pdb ID: 2EYI and 2R0O) these coiled regions interact via a *π*-cation bond between His170 and Arg232 (or His170 and Lys232 in pdb ID: 1TJT). In *S. pombe* the equivalent positions are Thr150 and Arg212 that do not interact, as the case also in human and mouse plectin.

**Figure 11:**
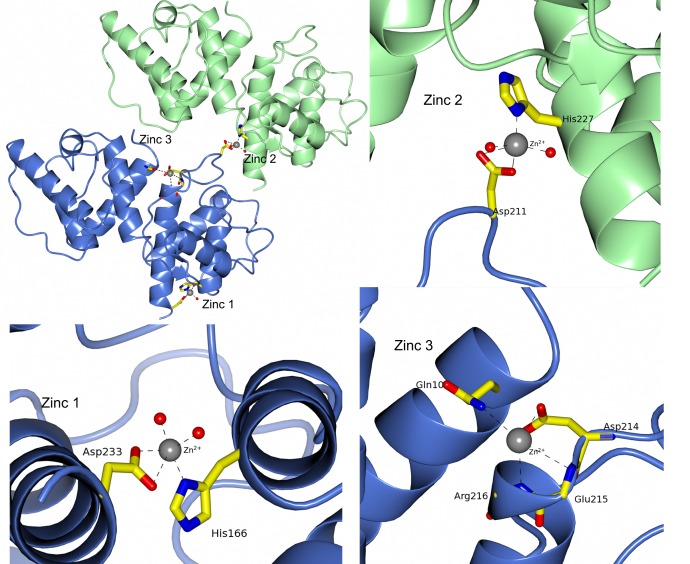
Zinc binding in *S. pombe* ADB. The presence of zinc was crucial for crystallization, hence three zinc ions have been included in the *S. pombe* ADB model. The first zinc is coordinated by the side chains of His166 and Asp233 and the second by the side chains of Asp211 and His227 from a symmetry related molecule. A third zinc, with low occupancy, has been modelled between the side chains of Asp214 and Gln10 and the main chain nitrogens of Glu215 and Arg216.

Three metal ions are found in the crystal structure and they are modelled as zinc due to the high concentration of zinc in the crystallization conditions. It should be pointed out, that we do not expect the presence of these zinc ions to have any biological function. The zinc ion with the highest occupancy is coordinated by His166 (ND1), Asp233 (OD1 and OD2) and two water molecules. A second zinc ion is coordinated by Asp211 (OD1 and OD2) and His227 (ND1) from a symmetry related molecule as well as two water molecules. A low occupancy zinc has been modelled, coordinated by Asp214 (OD1), Gln10 (NE2) and the main chain nitrogen of Arg216 (Fig. 11). Refined occupancies are 0.93, 0.81 and 0.38, respectively.

## Conclusions

We have cloned and characterized the structural domains of *S. pombe α*-actinin. The anomalous gel filtration behaviour indicated that also this *α*-actinin has an elongated shape and forms dimers. Further, we have also showed that the rod domain is required as well as sufficient for dimer formation, a prerequisite for actin cross-linking activity.

The results also indicated that *S. pombe α*-actinin is a proper actin cross-linker but it cross-links only non-muscle filamentous actin. The determined crystal structure of the actin-binding domain implied that the additional bonds observed between the two CH domains may stabilise the closed state. As this is the only significant difference between the actin-binding domain of *S. pombe* and that of human *α*-actinin3, this may explain why *S. pombe α*-actinin distinguishes between muscle and non-muscle actin. Whether this *α*-actinin belongs to the calcium-regulated isoforms or is calcium-insensitive requires further work.

## Additional Information and Declarations

### Data Availability

The following information was supplied regarding data availability: The Protein Data Bank: pdb ID 5BVR
